# Comparative Physicochemical Analysis of Reverse Osmosis-Treated Water and Natural Drinking Water Used by Patients With Chronic Kidney Disease of Unknown Etiology

**DOI:** 10.7759/cureus.107075

**Published:** 2026-04-15

**Authors:** Vigneswarran Arunachalam, Joseph Wilfred Amrit, Ponnazhagan K, Sumanth Kumar B

**Affiliations:** 1 Department of Biochemistry, Meenakshi Medical College Hospital and Research Institute, Meenakshi Academy of Higher Education and Research, Chennai, IND; 2 Department of Urology, Sri Muthukumaran Medical College Hospital, Chennai, IND

**Keywords:** chronic kidney disease of unknown etiology, hydrochemical exposure, natural drinking water, reverse osmosis, total dissolved solids

## Abstract

Background: Chronic kidney disease of unknown etiology (CKDu) is increasingly recognized in environmentally vulnerable regions where conventional causes do not fully explain disease burden. Drinking-water quality has been proposed as a relevant exposure. This study compared reverse osmosis-treated water and natural drinking water used in a CKDu-affected locality.

Methods: This exploratory comparative analytical observational study with a case-control framework was conducted in Kanchipuram, India, from December 2024 to October 2025. A total of 52 participants were included: 26 (50.0%) with CKDu and 26 (50.0%) non-CKDu controls. One participant-linked drinking-water sample was collected from each subject. Samples were analyzed for pH, total dissolved solids (TDS), sodium, potassium, anion gap, cadmium, copper, lead, zinc, and fluoride.

Results: Reverse osmosis-treated water showed markedly lower TDS than natural drinking water (151.03 ± 18.4 vs 662.63 ± 72.8 mg/L, p < 0.001). Cadmium, copper, lead, zinc, and fluoride were also significantly lower in reverse osmosis-treated water (all p < 0.001). pH differed slightly, but both groups remained near neutral. Sodium, potassium, and anion gap showed no significant differences.

Conclusion: Reverse osmosis-treated water and natural drinking water were chemically distinct exposures. The difference was driven mainly by TDS and trace-contaminant burden rather than routine electrolytes. These findings should be interpreted as exploratory and hypothesis-generating and support the relevance of local hydrochemical exposure in CKDu-affected settings.

## Introduction

Chronic kidney disease of unknown etiology (CKDu) has emerged as one of the most perplexing forms of nontraditional kidney disease in recent decades. Unlike conventional chronic kidney disease, which is usually anchored to diabetes mellitus, long-standing hypertension, or structural renal pathology, CKDu has been described largely in rural and agricultural communities where these classic drivers fail to fully account for the burden of disease. Contemporary reviews now portray CKDu as a geographically dispersed but epidemiologically uneven public health problem with reported hotspots across South Asia, Central America, Africa, and parts of the Middle East and with no single unifying etiological explanation yet accepted [1-3]. This uncertainty has shifted the scientific conversation away from monocausal thinking and toward a more layered framework in which occupational heat stress, dehydration, groundwater chemistry, agrochemical exposure, trace elements, and local ecological conditions may interact rather than act alone.

Within South Asia, the question of water has assumed particular importance. In Sri Lanka, India, and other endemic regions, drinking-water sources are not merely domestic utilities, but long-term environmental exposures embedded in livelihood geography, climate, and land use. Several investigations have shown that groundwater in affected settings may carry elevated total dissolved solid (TDS) hardness, and fluoride, nitrate, chloride, arsenic, or mixed ionic burdens, even when no singular nephrotoxin consistently emerges across all regions. In Sri Lanka, for example, groundwater studies and reviews have repeatedly described persistent elevations in hardness, alkalinity, ionicity, and selected contaminants in CKDu-prone areas while also emphasizing that spatial overlap does not by itself prove causation [4-6]. Similarly, in India, environmental studies from CKDu-affected or comparable hydrogeochemical settings have raised concern regarding acidic groundwater, elevated lead, silica hardness, fluoride salinity, and complex rock-water interaction patterns, although causal linkage to renal disease remains unresolved [7,8].

What has become increasingly clear is that the hydrochemical environment itself is heterogeneous and often dynamic. Studies from Tamil Nadu and adjoining regions have demonstrated substantial variability in pH, electrical conductivity, TDS hardness, and ionic composition across groundwater systems, depending on geology, season, salinity intrusion, and anthropogenic influence. Kumar et al. (2017) [9] reported TDS values ranging from 288 to 1600 mg/L in Tiruvallur with hydrochemical signatures shaped chiefly by rock-water interaction. Stanly et al. (2021) [10] found that coastal groundwater in southwest Tamil Nadu was spatially heterogeneous with mean TDS values of 495 and 445 mg/L across two seasons and evidence of saline influence in selected zones. Other regional surveys have shown bore well waters with markedly elevated salinity, hardness, alkalinity, and chloride burden or site-specific deterioration requiring treatment before domestic use [11,12]. Even where most samples are broadly classified as drinkable at the population level, localized departures from acceptable water quality remain common and may be environmentally meaningful.

Parallel to this hydrochemical literature is a second line of evidence that indirectly strengthens the relevance of the drinking-water source. Community behavior in endemic areas has shifted substantially toward treated water, particularly reverse osmosis water, because untreated groundwater is often perceived as unsafe. In Sri Lanka, nearly half of the surveyed groundwater-reliant households had transitioned to reverse osmosis water, and such water was rated as one of the safest available options by affected communities [13]. Ecological evidence has also suggested that expansion of reverse osmosis water access in endemic provinces coincided with a later decline in newly diagnosed CKD or CKDu cases in many administrative divisions, although such population-level observations remain insufficient to establish causality [14]. Operational studies further show that reverse osmosis systems can dramatically reduce excessive dissolved constituents even if maintenance recovery efficiency and disinfection remain ongoing challenges [15]. Taken together, these findings do not prove that reverse osmosis prevents CKDu, but they do indicate that the distinction between treated and untreated drinking water is epidemiologically and socially relevant. The primary objective of this exploratory study was to compare the physicochemical and biochemical characteristics of reverse osmosis-treated and natural drinking water in a CKDu-affected locality. The secondary exploratory objective was to assess whether the observed water-quality differences plausibly aligned with the environmental background in which CKDu had been observed locally.

## Materials and methods

This was a comparative analytical observational study with a case-control framework conducted at the Department of Biochemistry, Meenakshi Medical College Hospital and Research Institute, Kanchipuram, India, during the period from December 2024 to October 2025. This study was initiated after obtaining the approvals of IEC: MMCH&RI ICE/PG/11 Nov 2024, dated 27 Nov 2024. The study was conceived to examine whether the physicochemical and biochemical profile of locally consumed drinking water differed according to source type within a CKDu-affected setting, while also incorporating a non-CKDu comparison group from the same geographic locality. More specifically, the investigation compared reverse osmosis-treated water with natural drinking water obtained from the same Kanchipuram district area.

Study population

No formal a priori sample size calculation was performed because this study was designed as an exploratory comparative environmental analysis and included all eligible participant-linked habitual drinking-water sources available during the study period. This yielded 52 water samples in total, with 26 samples in each comparison group. Given this sample size, the study was expected to have reasonable ability to detect moderate-to-large between-group differences, whereas smaller differences may not have been detected reliably. Accordingly, the results should be interpreted as exploratory and hypothesis-generating rather than definitive.

Operational approach to CKDu classification

For the purpose of this study, CKDu was operationally understood as chronic kidney disease identified in the absence of overt conventional causes, particularly diabetes mellitus, long-standing hypertension, primary glomerular disease, obstructive uropathy, congenital renal disorders, or other known structural renal pathologies, insofar as such exclusions had been established in the underlying clinical records. This exclusion-based framing is consistent with the broader epidemiological literature on CKDu, in which the disorder is usually defined not by a single pathognomonic biomarker but by persistent renal dysfunction occurring outside the explanatory reach of traditional etiologies in vulnerable populations.

Inclusion and exclusion criteria

Participants were included if they had documented status as either CKDu or non-CKDu, resided within the study locality, and reported regular consumption of either reverse osmosis-treated water or natural drinking water from the corresponding area. Only participants whose principal drinking-water source could be identified and sampled during the study period were eligible for enrollment.

Patients with chronic kidney disease attributable to recognized causes, including diabetes mellitus, chronic hypertension, obstructive nephropathy, known glomerulonephritis, hereditary renal disease, or other established renal disorders, were excluded wherever such information was available from the clinical record. Individuals without a clearly identifiable habitual water source, or those for whom corresponding water sampling could not be completed, were also excluded.

Water sample collection

A total of 52 drinking-water samples were collected, with one sample obtained from each participant-linked habitual water source during the study period. Water sources were categorized as either reverse osmosis-treated drinking water or natural drinking water, based on the reported routine consumption pattern of the participants. Reverse osmosis-treated water samples were obtained from domestic point-of-use reverse osmosis purifiers. Samples were collected in sterile clean polyethylene containers between 9:00 AM and 1:00 PM and transported for laboratory analysis under standard handling conditions.

Physicochemical and biochemical analysis

All physicochemical measurements were performed using instruments maintained in calibrated condition according to routine laboratory standard operating procedures. The TDS meter and pH meter were calibrated before use with appropriate standard reference solutions, and calibration status was verified before measurement. Water samples were collected in sterile clean polyethylene containers, labelled immediately, capped without delay, and transported under standardized handling conditions to minimize preanalytical variation and external contamination. Uniform collection and analytical procedures were followed for all samples, and direct unnecessary transfer between containers was avoided as far as feasible. Standard precautions were observed during collection, handling, and analysis to reduce contamination risk. However, duplicate testing and formal blank-sample based contamination checks were not undertaken in the present study and should therefore be considered when interpreting the findings.

Outcome measures

The primary outcome of the study was the comparative physicochemical and biochemical characterization of natural drinking water and reverse osmosis-treated water. The secondary outcome was exploratory: to assess whether differences in measured water-quality parameters could plausibly align with the environmental background in which CKDu had been observed locally.

The present study was designed primarily for comparative characterization of participant-linked drinking-water sources and not as a comprehensive multivariable exposure-assessment study. Detailed information on potentially relevant confounding variables, including duration of habitual water consumption, occupational or agricultural exposure, heat-related work conditions, agrochemical contact, and dietary factors, was not systematically collected or adjusted for in the analysis. This approach was considered particularly important to acknowledge because existing CKDu literature suggests that the disorder is likely multifactorial, with reported associations involving farming-related occupations, rural environmental exposures, dehydration or heat stress, water-source characteristics, and contamination of food and water. Therefore, the measured differences in water-quality parameters should be interpreted as environmental observations within a CKDu-affected setting and not as independent causal determinants of disease.

Statistical analysis

All data were entered into a structured database and analyzed using IBM SPSS Statistics for Windows, Version 23 (Released 2015; IBM Corp., Armonk, New York, United States). Continuous variables were expressed as mean ± standard deviation. Normality of continuous variables was assessed using the Shapiro-Wilk test and visual inspection of distribution patterns. Variables with approximately normal distribution were compared using the independent-samples t-test, whereas non-normally distributed variables were compared using the Mann-Whitney U test. A p-value of less than 0.05 was considered statistically significant.

## Results

A total of 52 participants were included in the study, comprising 26 patients with CKDu and 26 non-CKDu controls from the same study locality. For each enrolled participant, one linked habitual drinking-water sample was collected and analyzed, yielding a total of 52 participant-linked water samples (Table 1).

**Table 1:** Distribution of study participants by disease status Descriptive distribution of enrolled participants according to disease status. CKDu: chronic kidney disease of unknown etiology.

Participant category	n	%
CKDu	26	50.0
Non-CKDu controls	26	50.0
Total	52	100.0

Physicochemical characteristics of reverse osmosis-treated and natural drinking water

Physicochemical analysis demonstrated a clear separation between reverse osmosis-treated water and natural drinking water, particularly with respect to TDS. The mean pH of reverse osmosis-treated water was slightly lower than that of natural drinking water, although both remained within a near-neutral range. This difference was statistically significant (7.46 ± 0.05 vs 7.52 ± 0.06; t = -3.92, p < 0.001). In contrast, TDS were markedly lower in reverse osmosis-treated water than in natural drinking water (151.03 ± 18.4 mg/L vs 662.63 ± 72.8 mg/L; Mann-Whitney U = 0, p < 0.001). Thus, although the pH difference reached statistical significance, the more environmentally meaningful distinction between the two water categories was the pronounced reduction in dissolved mineral burden after reverse osmosis treatment (Table 2).

**Table 2:** Physicochemical comparison of reverse osmosis-treated water and natural drinking water Data are presented as mean ± standard deviation. TDS: total dissolved solids. pH was compared using the independent-samples t-test, whereas TDS were compared using the Mann-Whitney U test.

Parameter	Reverse osmosis-treated water	Natural drinking water	Test statistic	p-value
pH	7.46 ± 0.05	7.52 ± 0.06	t = -3.92	<0.001
TDS (mg/L)	151.03 ± 18.4	662.63 ± 72.8	Mann-Whitney U = 0	<0.001

Heavy metals and fluoride burden

A similarly marked contrast was observed for selected heavy metals and fluoride. Natural drinking water showed consistently higher concentrations of cadmium, copper, lead, zinc, and fluoride than reverse osmosis-treated water. Cadmium levels were significantly higher in natural drinking water than in reverse osmosis-treated water (0.68 ± 0.05 µg/L vs 0.02 ± 0.006 µg/L; Mann-Whitney U = 0, p < 0.001). Copper also showed a marked elevation in natural drinking water (30.26 ± 24.91 µg/L vs 1.01 ± 0.21 µg/L; Mann-Whitney U = 81, p < 0.001). Lead concentrations were substantially greater in natural drinking water (8.39 ± 2.6 µg/L vs 0.42 ± 0.18 µg/L; Mann-Whitney U = 1, p < 0.001), as were zinc concentrations (1.06 ± 0.44 µg/L vs 0.02 ± 0.001 µg/L; Mann-Whitney U = 6, p < 0.001). Fluoride showed one of the largest between-group differences, with much higher levels in natural drinking water than in reverse osmosis-treated water (1800 ± 230 µg/L vs 90 ± 16 µg/L; Mann-Whitney U = 0, p < 0.001). Collectively, these findings indicate that the two water-source categories differed not only in general mineral load but also in trace-chemical burden (Table 3).

**Table 3:** Comparison of selected heavy metals and fluoride levels in natural drinking water and reverse osmosis-treated water Data are presented as mean ± standard deviation. Cd: cadmium; Cu: copper; Pb: lead; Zn: zinc; F⁻: fluoride. Intergroup comparisons were performed using the Mann-Whitney U test.

Variable	Natural drinking water (µg/L)	Reverse osmosis-treated water (µg/L)	Test statistic	p-value
Cadmium (Cd)	0.68 ± 0.05	0.02 ± 0.006	Mann-Whitney U = 0	<0.001
Copper (Cu)	30.26 ± 24.91	1.01 ± 0.21	Mann-Whitney U = 81	<0.001
Lead (Pb)	8.39 ± 2.6	0.42 ± 0.18	Mann-Whitney U = 1	<0.001
Zinc (Zn)	1.06 ± 0.44	0.02 ± 0.001	Mann-Whitney U = 6	<0.001
Fluoride (F⁻)	1800 ± 230	90 ± 16	Mann-Whitney U = 0	<0.001

Electrolytes and anion gap

In contrast to the marked differences seen in TDS and trace-chemical indices, the measured electrolyte-related variables were broadly similar between the two water-source categories. Sodium concentration did not differ significantly between reverse osmosis-treated water and natural drinking water (29.04 ± 4.72 mmol/L vs 29.81 ± 1.52 mmol/L; t = -0.79, p = 0.432). Potassium values were also nearly identical between the two groups (0.55 ± 0.03 mmol/L vs 0.54 ± 0.36 mmol/L; t = 0.14, p = 0.888). Likewise, the anion gap remained comparable across groups (30.64 ± 10.13 mmol/L vs 30.25 ± 7.47 mmol/L; t = 0.16, p = 0.875) (Table 4).

**Table 4:** Electrolytes and anion gap in reverse osmosis-treated water and natural drinking water Data are presented as mean ± standard deviation. Intergroup comparisons were performed using the independent-samples t-test.

Parameter	Reverse osmosis-treated water	Natural drinking water	Test statistic	p-value
Sodium (mmol/L)	29.04 ± 4.72	29.81 ± 1.52	t = -0.79	0.432
Potassium (mmol/L)	0.55 ± 0.03	0.54 ± 0.36	t = 0.14	0.888
Anion gap (mmol/L)	30.64 ± 10.13	30.25 ± 7.47	t = 0.16	0.875

## Discussion

The present study shows a clear hydrochemical divide between reverse osmosis-treated water and natural drinking water in a CKDu-affected setting. The most important finding was not a trivial shift in one isolated analyte, but a broad reduction in dissolved and potentially nephrotoxic burden in the RO-treated samples. TDS were dramatically lower in RO water than in natural water, and the same directional pattern was seen for cadmium, copper, lead, zinc, and fluoride, all of which were substantially reduced after treatment. By contrast, sodium, potassium, and anion gap showed no meaningful difference between the two source categories. This pattern is important because it suggests that the environmental signal in this locality is carried less by simple electrolyte variation and more by the combined load of dissolved solids, trace elements, and fluoride. The small difference in pH, although statistically significant, remained within a near-neutral range and is unlikely to be the central biologic issue. The more persuasive signal is therefore compositional burden rather than acidity alone.

This interpretation is broadly consistent with the wider CKDu literature, which increasingly argues against a single-agent model and instead points toward a composite hydrochemical environment. Reviews by Priyadarshani et al. (2023) [1], Rao et al. (2023) [2], Bradley et al. (2025) [3], and Hettithanthri et al. (2021) [6] all converge on the view that CKDu likely emerges from interacting exposures rather than one exclusive toxin. The current findings fit that framework well. Natural water in the study area had markedly higher TDS and fluoride, and this resonates with observations from Sri Lankan endemic regions where persistent ionicity, hardness, and fluoride enrichment have repeatedly been reported as environmentally plausible contributors. Imbulana et al. (2021) [4] found poor potability with high TDS and hardness across seasons, while Gobalarajah et al. (2020) [16] linked higher TDS and arsenic with worse renal indices. Piyathilake et al. (2021) [17] similarly identified fluoride as one of the strongest geochemical correlates of CKDu presence at source level. The current study does not test disease prediction directly, yet its chemical profile sits comfortably within that broader epidemiological landscape.

The findings also align with Indian groundwater studies showing that water quality in Tamil Nadu and adjoining coastal belts is highly heterogeneous, often with problematic salinity, hardness, and mineral burden. Kumar et al. (2017) [9], Stanly et al. (2021) [10], Sivakumar et al. (2023) [11], and Promilton et al. (2025) [12] all documented substantial spatial variation in TDS and ionic composition, with a considerable fraction of samples falling into categories that would merit treatment before drinking. In that sense, the natural water profile observed here is not chemically implausible or anomalous for the region. Rather, it appears to reflect a recognizable groundwater signature shaped by rock-water interaction, evaporation concentration, and local anthropogenic influence.

At the same time, some elements of the present findings do not fully mirror the published literature, and those differences deserve careful interpretation. Several reports have emphasized hardness, magnesium, nitrate, phosphate, arsenic, silica, agrochemicals, or organic contaminants as potentially relevant exposures, whereas the present study did not assess many of these variables. Lal et al. (2020) [7] highlighted acidic groundwater, silica, and lead in Uddanam; Gobalarajah et al. (2020) [16] underscored arsenic; Piyathilake et al. (2021) [17] pointed to phosphate in addition to fluoride; and Vlahos et al. (2021) [18] detected agrochemicals in a large proportion of wells. The absence of these measurements here does not negate those pathways. It simply means that the current dataset captures only one segment of the environmental puzzle. Likewise, some studies have found no convincing independent role for certain heavy metals, particularly when exposure is examined more rigorously or across multiple pathways. Kulathunga et al. (2022) [19], for example, did not support cadmium, arsenic, and lead in drinking water as primary drivers, despite identifying chronic dietary lead concern. Such disagreement is not surprising. CKDu research is methodologically fragmented, case definitions vary, exposure matrices differ, and local hydrogeology can sharply alter which contaminants dominate.

From a clinical and public health perspective, the present findings support a pragmatic rather than ideological message. Even without proving causation, the study shows that RO treatment materially lowers the dissolved contaminant burden of drinking water in this locality. That alone has preventive relevance in a disease landscape where safe water access is repeatedly proposed as a sensible intervention. The ecological observations of Ranasinghe et al. (2024) [14] and the behavioral work of Horbulyk et al. (2021) [13] suggest that communities recognize this logic and often move toward treated water when trust and access improve. For clinicians working in CKDu-prone areas, these data reinforce the importance of environmental history taking, especially habitual water source, and support collaboration with public health agencies on water surveillance rather than restricting CKDu care to late-stage renal management.

The study has important limitations. The sample size was modest, which limits statistical precision and generalizability. The design was observational and cross-sectional, so temporal sequence and causality cannot be established. Only participant-linked water chemistry was assessed; no biomarker correlation, dose-response analysis, or longitudinal renal outcome was performed, which restricts direct clinical interpretation. Although the case-control framework improves contextual relevance, the reported results primarily compare water categories rather than directly linking specific water exposures to CKDu odds or severity. Seasonal variability was not examined, and the analyte panel was incomplete, excluding hardness, calcium, magnesium, nitrate, arsenic, silica, and agrochemical residues, thereby limiting exposure completeness. Finally, a single sampled source may not perfectly represent long-term cumulative exposure.

## Conclusions

This study demonstrates that reverse osmosis-treated drinking water and natural drinking water in a CKDu-affected locality are not chemically interchangeable exposures. The principal distinction was not a marginal shift in routine electrolytes, but a marked reduction in TDS and selected trace contaminants, particularly cadmium, lead, copper, zinc, and fluoride, in the reverse osmosis-treated samples. These findings do not establish a direct causal pathway between water chemistry and CKDu, but they support the relevance of local hydrochemical exposure as an environmental consideration in CKDu-affected settings. Reverse osmosis treatment was associated with a substantially lower composite contaminant burden in the sampled drinking-water sources; whether this translates into reduced renal risk requires larger longitudinal studies with broader contaminant profiling and clinical correlation. The present findings should therefore be interpreted as exploratory and hypothesis-generating.
